# High prevalence of mutation in the *Plasmodium falciparum dhfr* and *dhps* genes in field isolates from Sabah, Northern Borneo

**DOI:** 10.1186/1475-2875-12-198

**Published:** 2013-06-12

**Authors:** Noor Rain Abdullah, Nor Azrina Norahmad, Jenarun Jelip, Lokman Hakim Sulaiman, Hasidah Mohd Sidek, Zakiah Ismail, Harald Noedl

**Affiliations:** 1 Herbal Medicine Research Centre, Institute for Medical Research, Kuala Lumpur 50588, Malaysia; 2 Sabah State Health Department, Rumah Persekutuan, Jalan Mat Salleh, Kota Kinabalu 88590, Sabah, Malaysia; 3 Office of the Deputy Director General of Health (Public Health), Ministry of Health Malaysia, Level 12 Block E7 Complex E, Federal Government Administrative Centre, Putrajaya 62590, Malaysia; 4 School of Biosciences and Biotechnology, Faculty of Science & Technology, Universiti Kebangsaan Malaysia, 43600, Bangi, Malaysia; 5 Institute of Specific Prophylaxis and Tropical Medicine, Medical University of Vienna, Kinderspitalgasse 15, Vienna A-1090, Austria

**Keywords:** Plasmodium falciparum, Sulphadoxine–pyrimethamine, Molecular markers

## Abstract

**Background:**

Sulphadoxine-pyrimethamine (SP) has been in use for the treatment of uncomplicated falciparum malaria in Malaysia since the 1970s and is still widely employed in spite of widespread clinical resistance. Resistance to SP is known to be mediated by mutations in the *pfdhfr* and *pfdhps* genes. The aim of the present study was to investigate the distribution of *pfdhfr* and *pfdhps* gene polymorphism in *Plasmodium falciparum* field isolates from Kalabakan, Sabah, in northern Borneo.

**Methods:**

A total number of 619 individuals were screened from 23 study sites of which 31 were positive for *P. falciparum.* Analysis of restriction fragment length polymorphisms (RFLP) was used to identify polymorphism in the *pfdhfr* and *pfdhps* genes at positions 16, 51, 59, 108, 164 and 437, 540, 581, respectively.

**Results:**

All samples had at least one mutation in each of the genes associated with drug resistance. The prevalence of *pfdhfr* 59arg, 164leu and 108asn were 100%, 80.65% and 58.06%, respectively. *Pfdhps* mutants 437gly and 581gly accounted for 100% and 74.19% respectively. In *pfdhfr*, the most common mutant genotypes were combination 59arg + 164leu (22.58%) and 59arg + 108asn + 164leu (51.61%). In *pfdhps* the most common genotype was 437gly + 581gly (74.19%). One individual (3.22%) harboured parasites with four *pfdhfr* (16 val + 59arg + 108asn + 164leu) and two *pfdhps* (437gly + 581gly) mutations. The highest quintuple *pfdhfr/pfdhps* (41.94%) was three *pfdhfr* (59arg + 108asn + 164gly) and two *pfdhps* (437gly + 581gly).

**Conclusion:**

The data suggest a high prevalence of genetic variations conferring resistance to SP which can predict treatment failure before becoming clinically evident. In areas like this, the use of SP may no longer be indicated.

## Background

Malaysia is in the pre-elimination phase of the malaria elimination programme [1]. Malaysia is fully committed to controlling malaria, providing good infrastructure for control which dates back to the programme’s implementation in 1961. The impact of the malaria eradication programme has resulted in a major reduction of malaria cases from 243,870 in 1961 to 44,226 in 1980 [1,2] and has provided the basis for the subsequent malaria control programme which began in 1982. In 2010, the National Malaria Control Programme was re-oriented from control to elimination, with the implementation of the “Malaria Elimination Programme”. This is in line with the global vision of malaria elimination and the aim of achieving malaria elimination status in Peninsular Malaysia by 2012 and Malaysian Borneo by 2020 [3,4].

The majority of the malaria cases reported in Malaysia in 2012 originated from two states in northern Borneo, Sabah (45%) and Sarawak (29%) (Vector Borne Disease Control 2012, unpublished data). This can largely be attributed to inaccessibility, climate and migration across the nearby border with Indonesia and results in a highly heterogeneous distribution of vector and parasite species as well as drug resistance patterns.

Drug resistance remains a major obstacle to malaria elimination efforts in the region [5,6]. Sulphadoxine-pyrimethamine (SP) has been in use for the treatment of uncomplicated falciparum malaria in Malaysia since the 1970s. It is used in remote areas for uncomplicated chloroquine-resistant *Plasmodium falciparum* infections for outpatient as well as inpatient malaria cases. In spite of clinical resistance reported in some places, this combination is still widely employed [7-10].

The genetic background of SP resistance is better documented than any other anti-malarial drug. Mutations in the dihydropteroate synthase (*dhps*) and dihydrofolate reductase (*dhfr*) genes, both coding for essential enzymes in the folate biosynthesis pathway, mediate drug resistance to SP [11-13]. *Pfdhfr* codon ser108asn is likely to play a key role in pyrimethamine resistance with mutations at 51ile, 59arg and 164 leu modulating the level of resistance [14]. The *pfdhfr* triple mutation 51ile, 59arg and 108asn, has been shown to be associated with SP treatment failure, regardless of *pfdhps* genotype. Study have shown that findings *in vitro* may have indication or consistent with reports of failure treatment in the country [15-17]. Sulphadoxine resistance in *P. falciparum* is associated with mutations at five *pfdhps* codons; 436ala/phe, 437gly, 540glu, 581gly and 613ser [18-20]. A strong indicator for SP treatment failure is the quintuple mutations in three *pfdhfr* codons (108asn + 51ile + 59arg) and two *pfdhps* codons (437gly + 540glu) [21,22].

In spite of a massive reduction in malaria cases in Malaysia, the drug resistance situation remains poorly documented [9]. Particularly in times of pre-elimination, a better understanding of the epidemiology of drug resistance has become vital for the region. The aim of the present study was therefore to investigate the distribution of *pfdhfr* and *pfdhps* gene polymorphism in *P. falciparum* field isolates from Kalabakan, Sabah, in northern Borneo.

## Methods

### Study area and sample collection

The study was conducted between 2008 and 2009 in Kalabakan, 100 km from Tawau, in Sabah, bordering East Kalimantan. Samples were collected by active case detection in villages, logging and road construction camps within a radius of 50 to 80 km from Kalabakan. Kalabakan contributes most of the malaria cases in Sabah and was reported to have the highest number of malaria cases in 2008 and 2009 with 21.54% and 22.79%, respectively, of the total number of cases in Sabah. At the time this study was conducted, SP was the official first-line treatment for uncomplicated falciparum malaria and it remains widely used.

The study protocol was reviewed and approved by the institutional review board of the Institute for Medical Research, Kuala Lumpur and the Medical Review and Ethics Committee of the Ministry of Health, Malaysia. Individuals who consented to participate in the study were screened for malaria using rapid diagnostic tests (RDT) (Paramax- 3TM; Zephyr Biomedicals, India). In addition, blood films for malaria parasite (BFMP) were prepared to determine parasite density. All study participants diagnosed positive for malaria infection by RDT had 500 μl of whole blood collected by venepuncture. Blood was then spotted on 3MM Whatman filter paper. The filter paper was allowed to dry completely, transferred into individual plastic bags, labelled, and transported to the Institute for Medical Research in Kuala Lumpur where confirmation of species by PCR and genotype analysis was conducted. Speciation for *Plasmodium vivax*, *P. falciparum, Plasmodium malariae* and *Plasmodium knowlesi* was undertaken using a modified version of published method [23,24].

### DNA extraction

DNA from filter paper was extracted using QIAampTM DNA mini kit (QIAmp; QIAGEN, Hilden, Germany), according to the manufacturer’s instructions (dried blood spots protocol) with the only modification being an adjustment of the elution buffer volume used to elute the DNA. DNA samples were then kept at −20°C until further processing.

### Genotyping of *pfdhfr* and *pfdhps* by PCR-RFLP

PCR reaction and the restriction fragment polymorphism protocol (PCR-RFLP) were used for the detection of mutation on *pfdhfr* as described elsewhere [25] with some modification to DNA and primer concentrations. The first round PCR reaction mixture consisted of 50 ng of genomic DNA, 0.20 μM of each primer, 200 μM dNTPs, 1.5 mM MgCl_2_, and 2.5 U of Taq polymerase in a final reaction volume of 50 μl. Two microlitres of the amplified product from the first PCR were subjected to two sets of secondary round PCR reaction mixtures containing 0.20 μM of each primer, 200 μM dNTPs, 2.0 mM MgCl2, and 2.5 U Taq polymerase. The PCR reaction used primers M4-F amplifying a 326 basepairs (bp) fragment containing cys59arg, ser108asn and ser108thr, and primers M3-F/amplifying a 522 bp fragment containing ala16val, asn51ile, ser108asn and ile164leu (Table 1).

**Table 1:** **The primer pairs, the cycling temperature and restriction enzymes used in detection of gene polymorphism on *pfdhfr***

**Second-round PCR for *pfdhfr *region containing the polymorphism**	**Cycling temperature**	**Size (bp)**	**To detect mutatin at codon:**	**Restriction enzyme**	**Fragment length (bp) Wild Type**	**Fragment length (bp) Mutant**
F: 5′-GAA-	94°C for 2 min;	326	cys	*XmnI*	189. 163	26, 137,
ATG-TAA-TTC-	94°C for 1 min,		59			163,
CCT-AGA-TAT-	45°C for 1 min,		arg			
GGA-ATA-TT-3′	72°C for 1 min			*BstNI*	326	145,181
	(5 cycles)		ser			
M4: 5′-TTA-			108			
ATT-TCC-CAA-	continue with		thr	*AluI*	118, 180	299
GTA-AAA-CTA-	40 cycles,					
TTA-GAG-	94°C for 1 min,		ser			
CTTC-3′	45°C for 1 min,		108			
	72°C for 1 min Further extension 72°C for 10 min		asp			
M3: 5′-TTT-	Same cycle as	522	ala	*NlaIII*	53, 93, 376	146, 245
ATG-ATG-GAA-	F-M4 PCR		16			
CAA-GTC-TGC-GAC-GTT- 3′			val			
			asn	*Tsp5091*	55, 65, 120,	55, 65, 120,
F/: 5′-AAA-TTC-			51		153	218
TTG-ATA-AAC-			ile			
AAC-GGA-ACC-						
TTT-TA-3′						
			ser	*BsrI*	522	190, 332
			108			
			asn			
			ile	*Dra I*	107, 171,	107, 143,
			164		245	245
			leu			

The fragment sizes of wild type and mutant are indicated.

Similarly, nested PCR was conducted for the detection of mutation in the *pfdhps* gene as described previously [25] with modification on the cycling temperature for the second-round PCR, DNA and primers concentration. The first round PCR was performed using the primer sets R1-R2, followed by two sets of second-round PCR using K-K/and L-L/primers pair (Table 2). The PCR reaction used primers K-K/amplifying a 438 bp fragment containing ala437gly and lys540glu, and primers L-L/amplifying a 161 bp fragment containing ala581gly. The PCR and the nested PCR reactions used the same final concentrations as in the first round PCR carried out for *pfdhfr*.

**Table 2:** **The primer pairs, the cycling temperature and restriction enzymes used in detection of gene polymorphism on *pfdhps***

**Second-round PCR for *pfdhps *region containing the polymorphism**	**Cycling temperature**	**Size (bp)**	**To detect mutation at codon**	**Restriction enzyme**	**Fragment length (bp) wild type**	**Fragment length (bp) mutant**
K: 5′ TGC-TAG-	94°C for 3 min	438	ala	*Ava II*	438	404
TGT-TAT-AGA-			437			
TAT-AGG-ATG-	40 cycles of		gly			
AGC-ATC-3′	94°C for 1 min					
	at 45°C for 45		lys	*FokI*	438	85, 320
K/: 5′-CTA-	sec, 72°C for 1		540			
TAA-CGA-GGT-ATT-GCA-TTT-AAT-GCA-AGA-A-3′	min, further extension 72°C for 10 min		glu			
L: 5′-ATA-	94°C for 2 min,	161	ala	*BstUI*	105	138
GGA-TAC-TAT-	45°C for 2 min,		581			
TTG-ATA-TTG-	72°C for 1 min		gly			
GAC-CAG-	30 sec (5 cycles)					
GAT-TCG-3′ L/: 5′-TAT-TAC-AAC-ATT-TTG-ATC-ATT-CGC-GCA-ACC-GG-3′	followed by 35 cycles 94°C for 1 min; 45°C for 1 min, 72°C for 1 min 30 sec.					
	Further extension at 72°C for 10 min					

The fragment sizes of wild type and mutant are indicated.

The products of the secondary PCR containing the polymorphic region were subjected to enzyme digestion for the detection of mutations at the various sites. The enzyme digestions were conducted according to manufacturer’s instructions (New England Biolabs, Beverly, MA). The details of primer sequences, cycling temperatures, restriction enzyme digestion and fragment sizes for each codon are shown in Tables 1 and 2. DNA of laboratory strain *P. falciparum* 3D7, K1, W2 and T9.96 were included in each reaction of PCR and RLFP and served as positive and negative controls. Water was used to replace the DNA template for the negative control.

### Analysis of PCR-RFLP products using the Agilent 2100 Bioanalyzer

The PCR-RFLP products were analysed using the Agilent 2100 Bioanalyzer and the Agilent DNA 1000 Kit (Agilent Technologies, Molecular Probes Inc, USA). The procedures were conducted according to manufacturer’s instructions (Agilent Technologies, Molecular Probes Inc, USA). The results were then viewed and analysed using the Agilent 2100 software.

## Results

### Sample collection

A total number of 619 individuals were enrolled and screened at 23 sites. Fifty-eight (9.37%) (95% [CI] = 7.07-11.67%) tested positive for malaria, 5% (95% [CI] = 3.28-6.72%) were positive for *P. falciparum*. The *pfdhfr* and *pfdhps* gene were successfully amplified on all the 31 samples from Kalabakan. These samples were then included in PCR-RFLP analysis for the determination of the prevalence of mutations in the *pfdhfr* and *pfdhps* genes.

### *Pfdhfr* mutant genotype

**Figure 1:**
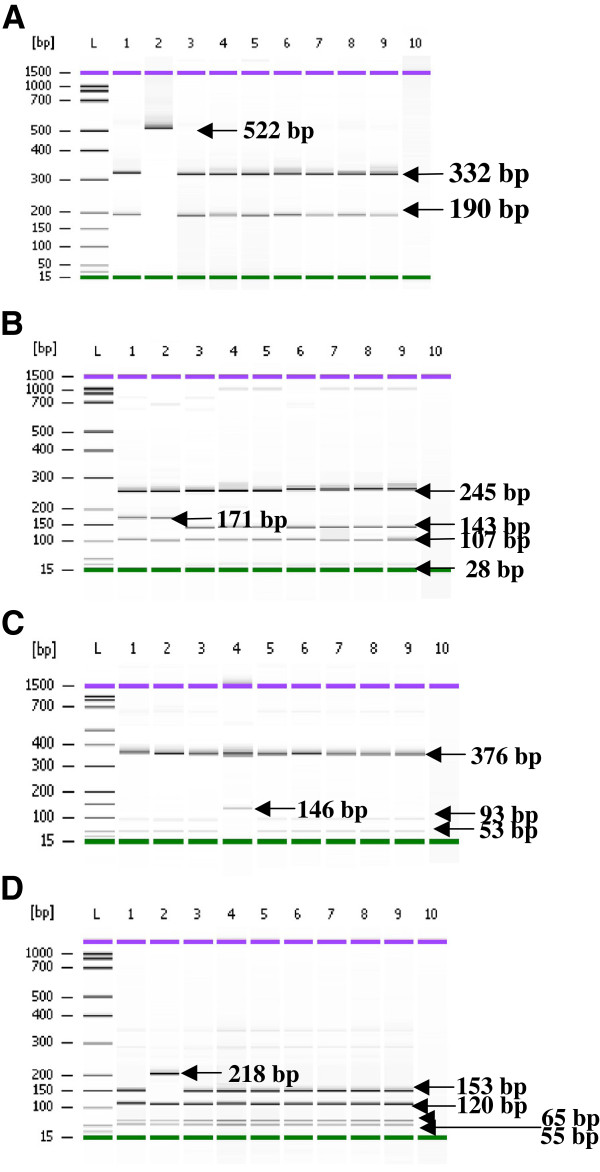
**PCR-RFLP of the *pfdhfr *gene, amplified region of M3-F/primers.***BsrI* cleaved the 522 bp fragment into 190 and 332 bp indication of 108asn mutation **(A)**. *DraI* detect 164leu mutations producing 28, 107, 171 and 245 bp fragments for wild type and 107, 143 and 245 bp for mutant **(B)**. Digestion with *NlaIII* produce 53, 93 and 376 bp for wild type and 146 and 245 bp for mutant at codon 16 **(C)** and digestion with Tsp5091 yielded 55, 65, 120, and 153 bp fragments for wild type and 55, 65, 120 and 218 bp fragments for mutant for detection on polymorphism at codon 51 **(D)**. Lane L: DNA ladder of Agilent DNA 1000 Kit (Agilent Technologies, Molecular Probes Inc, USA), The controls are in Lane 1 and 2: **A:** Lane 1: *Plasmodium falciparum* KI strain (mutant); Lane 2: *P. falciparum* T9.96 strain (wild type); Lane 3–9: field samples from Kalabakan; well 10: PCR negative control (no DNA was added to the PCR reaction). **B** and **C**: Lane 1: *Plasmodium falciparum* KI strain (wild type); Lane 2: *P. falciparum* T9.96 strain (wild type); Lane 3–9: field samples from Kalabakan; well 10: PCR negative control (no DNA was added to the PCR reaction). **D**: Lane 1: *Plasmodium falciparum* KI strain (wild type); Lane 2: *P. falciparum* W2 strain (mutant) Lane 3–9: field samples from Kalabakan; well 10: PCR negative control (no DNA was added to the PCR reaction).

**Figure 2:**
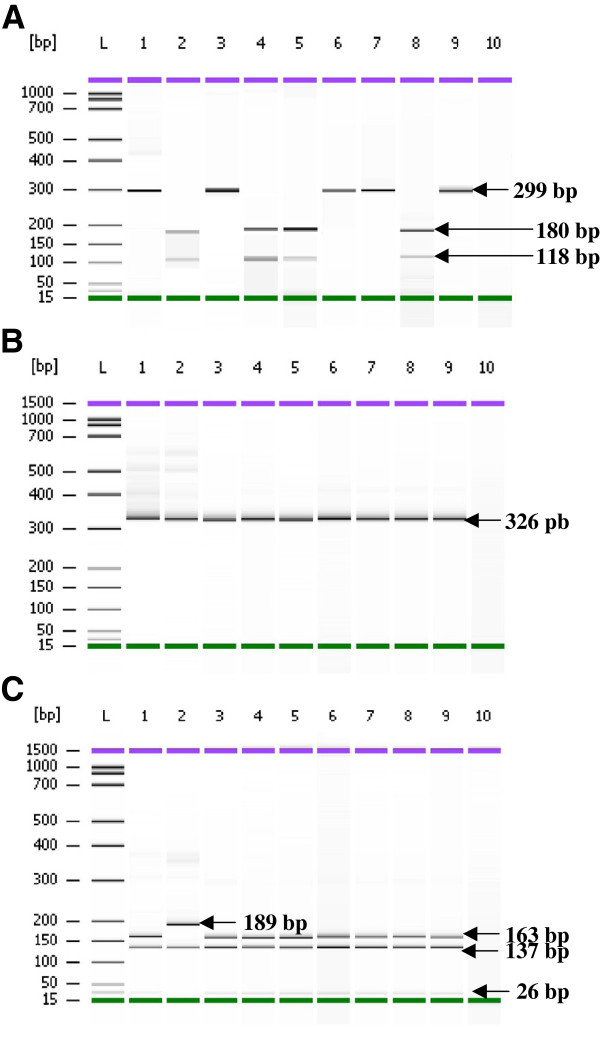
**PCR-RFLP of the *pfdhfr *gene, amplified region of M4-F primers.** The 326 bp fragment was cut by *Alu I* into 180 and 118 bp fragments, indications for wild type ser108 and 299 bp for mutant **(A)**. *BstNI* digested only mutant allele into 145 and 181 bp indicates mutation 108thr. All the tested samples showed wild type **(B)**. Reaction with *XmnI* produced fragment 163 and 189 bp for wild type; and 26, 137 and 163 bps for 59arg mutation **(C)**. Lane L: DNA ladder of Agilent DNA 1000 Kit (Agilent Technologies, Molecular Probes Inc, USA), The controls are in Lane 1 and 2: **A**: Lane 1: *Plasmodium falciparum* KI strain (mutant); Lane 2: *P. falciparum* T9.96 strain (wild type); Lane 3–9: samples from Kalabakan; Lane 10: PCR negative control (no DNA was added to the PCR reaction). **B**: Lane 1: *Plasmodium falciparum* KI strain (wild type); Lane 2: *P. falciparum* T9.96 strain (wild type); Lane 3–9: samples from Kalabakan; Lane 10: PCR negative control (no DNA was added to the PCR reaction). **C**: Lane 1: *Plasmodium falciparum* KI strain (mutant); Lane 2: *P. falciparum* T9.96 strain (wild type); Lane 3–9: samples from Kalabakan; Lane 10: PCR negative control (no DNA was added to the PCR reaction).

PCR and RFLP products were analysed by Agilent 2100 Bioanalyzer and the Agilent DNA 1000 Kit for the detection of mutations in the *pfdhfr* gene (details in Figures 1 and 2). Based on PCR-RFLP findings 100%, 80.64% (95% [CI] = 66.74-94.56%), and 58.06% (95% [CI] = 40.69-75.43%) were classified as *pfdhfr* mutants 59arg, 164leu and 108asn, respectively (Table 3). Mutation at codon 164 and 108 has been identified as strong determinant for pyrimethamine resistant. No mutation was detected at codon 51.

**Table 3:** PCR-RFLP* findings of polymorphism on the *pfdhfr *and *pfdhps *gene from samples (n = 31) collected in Kalabakan, Sabah

	***pfdhfr***						***pfdhps***		
Codons	ala	asn	cys	ser	ser	ile	ala	lys	ala
	16	51	59	108	108	164	437	540	581
	val	ile	arg	asn	thr	leu	gly	glu	gly
% Mutation	16.12	0	100	58.06	0	80.64	100	0	74.19
Wild-Type	83.87	100	0	49.94	100	19.35	0	100	25.8

* PCR-RFLP, polymerase chain reaction-restriction fragment length polymorphism

§ Alanine = ala, Valine = val, Asparagine = asn, Isoleucine = ile, Cysteine = cys, Arginine = arg, Serine = ser, Threonine = thr, Leucine = leu, Glycine = gly, Lysine = lys, Glutamic acid = glu.

### *Pfdhps* mutant genotype

**Figure 3:**
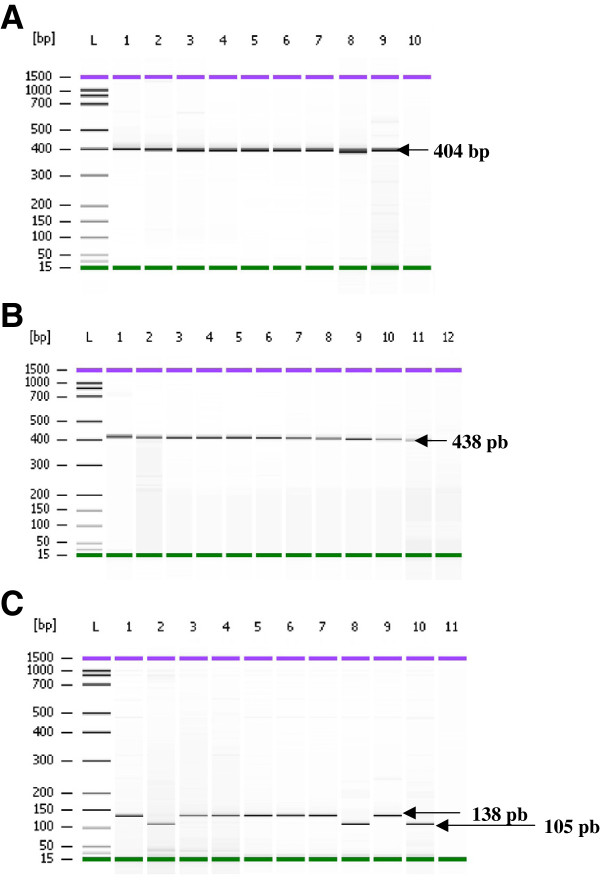
**PCR-RFLP of *pfdhps *gene.** Amplified region of K-K/primers was targeted to ala437gly with *AvaII* and lys540glu with *FokI*, showed 404 bp (mutant) and 438 bps (wild type) respectively **(A, B)**. Reaction of L-L/amplified region with *BstUI* showed presence of 581gly mutation with a 138 bp fragment and wild type ala581 producing a 105 bp **(C)**. Lane L: DNA ladder of Agilent DNA 1000 Kit (Agilent Technologies, Molecular Probes Inc, USA). The controls are in Lane 1 and 2: **A**: Lane 1: *Plasmodium falciparum* 3D7 strain (mutant); Lane 2: *P. falciparum* K1 strain (mutant); **B**: Lane 1: *Plasmodium falciparum* K1 strain (wild type); Lane 2: *P. falciparum* T9.96 strain (wild type); **C**: Lane 1: *Plasmodium falciparum* K1 strain (mutant); Lane 2: *P. falciparum* W2 strain (wild type). Lane 3–10: samples from Kalabakan; Lane 11: PCR negative control (no DNA was added to the PCR reaction).

The most common mutations of the *pfdhps* gene (details in Figure 3) were observed at 437gly (100%) and 581gly (74.19%) (95% [CI] = 58.79–89.59%) which are commonly associated with sulphadoxine resistance (Table 3). No mutation was observed at codon 540.

### *Pfdhfr/pfdhps* mutant genotype combination

Five different mutant genotypes related to the *pfdhfr* gene (Table 4) were identified. The three most common combinations were 59arg + 108asn + 164leu (51.61%) (95% [CI] = 34.02%-69.2%) and 59arg + 164leu (22.58%) (95% [CI] = 7.86%-37.3%). The rest of the genes, 25.81% (95% [CI] = 10.41% to 41.21%), existed either on its own or in combination with the other genes (Table 4). *Pfdhps* mutant genotypes combined 437gly + 581gly (74.19%) (95% [CI] = 58.79%-89.59%) (Table 4). When combining *pfdhfr* and *pfdhps* mutations, there was one parasite isolate (3.22%) (95% [CI] = −2.99%-9.43%) harbouring four *pfdhfr* (16 val + 59arg + 108asn + 164leu) and two *pfdhps* (437gly + 581gly) mutations (Table 5). The most commonly found mutant genotype combining *pfdhfr* and *pfdhps* linked three *pfdhfr* mutations 59arg + 108asn + 164leu with two *pfdhps* mutations 437gly + 581gly with 41.94% (95% [CI] = 24.57%- 59.31%) (Table 5).

**Table 4:** **Frequency of mutant genotype for the *pfdhfr *and *pfdhps *genes in samples from Kalabakan, Tawau**

**Gene**	**Mutant genotype**	**% (95% [CI])**
*pfdhfr*	59arg	6.45 (95% [Cl] = −2.2-15.1%)
	16 val, 59arg	9.67 (95% [Cl] = −0.73-20.07%)
	59arg, 108asn	3.23 (95% [Cl] = −2.99-9.45%)
	59arg, 164leu	22.58 (95% [Cl] = 7.86-37.3%)
	59arg, 108asn, 164leu	51.61 (95% [Cl] = 34.02-69.2%)
	16 val, 59arg, 164leu	3.23 (95% [Cl] = −2.99-9.45%)
	16 val, 59arg, 108asn, 164leu	3.23 (95% [Cl] = −2.99-9.45%)
*pfdhps*	437gly, 581gly	74.19 (95% [CI] = 58.79 – 89.59%)

*Valine = val, Asparagine = asn, Arginine = arg, Leucine = leu, Glycine = gly.

**Table 5:** **The frequency of mutant genotypes combining mutations in the *pfdhfr *and *pfdhps *genes in isolates collected during the study in Kalabakan (n = 31)**

***Pfdhfr codons***						***Pfdhps codons***			***Frequency of mutant genotype***	
ala 16 val	asn 51 ile	cys 59 arg	ser 108 asn	ser 108 thr	ile 164 leu	ala 437 gly	lys 540 glu	ala 581 gly	% of mutant genotype in sample	*dhfr/dhps* combination
***val***	asn	***arg***	***asn***	ser	***leu***	***gly***	Lys	***gly***	3.22 (1/31)	4dhfr, 2 dhps
***val***	asn	***arg***	ser	ser	***leu***	***gly***	Lys	***gly***	3.22 (1/31)	3dhfr, 2 dhps
ala	asn	***arg***	***asn***	ser	***leu***	***gly***	Lys	***gly***	41.94 (13/31)	3dhfr, 2 dhps
***val***	asn	***arg***	ser	ser	ile	***gly***	Lys	***gly***	9.67 (3/31)	2dhfr, 2dhps
ala	asn	***arg***	ser	ser	***leu***	***gly***	Lys	***gly***	16.12 (5/31)	2dhfr, 2dhps
ala	asn	***arg***	ser	ser	ile	***gly***	Lys	***gly***	3.22 (1/31)	1dhfr, 2dhps
ala	asn	***arg***	ser	ser	ile	***gly***	Lys	ala	3.22 (1/31)	1dhfr, 1dhps
ala	asn	***arg***	ser	ser	***leu***	***gly***	Lys	ala	6.45 (2/31)	2dhfr, 1dhps
ala	asn	***arg***	***asn***	ser	***leu***	***gly***	Lys	ala	12.90 (4/31)	3dhfr, 1dhps

* Alanine = ala, Valine = val, Asparagine = asn, Isoleucine = ile, Cysteine = cys, Arginine = arg, Serine = ser, Threonine = thr, Leucine = leu, Glycine = gly, Lysine = lys, Glutamic acid = glu.

§ Mutation is represented by amino acid in italic bold.

## Discussion

The study report the prevalence of mutations in the *pfdhfr* and *pfdhps* genes in *P. falciparum* field isolates collected from individuals in Kalabakan, Sabah (Northern Borneo). This region remains the focus of *P. falciparum* infection in a country that has reached pre-elimination and is striving for malaria elimination in the coming years. SP has been used extensively in the region for more than 30 years, as first-line drug until 2010 [26]. This is surprising as SP treatment failures have been reported from Malaysia as early as 1982 [7]. By the late 1990s, SP resistance had reached 47.4% in Peninsular Malaysia [8] and 29.4% in Tawau, Sabah [9] and first evidence of the molecular background of SP resistance in Malaysia came from a study conducted on mainland Malaysia and Borneo reported in 2001 [27].

The findings of the study indicate that all samples collected harbour at least one of the markers known to be involved in SP resistance on both the *pfdhfr* as well as the *pfdhps* gene. Without exception all samples (100%) had the *pfdhps* mutation 437gly, which is a common observation in areas where SP is widely used. Earlier studies suggest that its presence alone or in combination with 540glu is predictive of early SP treatment failure [28,29]. The 540glu is typically found together with 437gly, particularly in Africa [30,31]. However, the findings of the study showed that all the samples harboured the *pfdhps* 437gly mutation together with 581 gly (74.19%) rather than 540glu. In fact, the studies indicate the complete absence of the 540glu mutation in the patient samples. Both combinations have been associated with sulphadoxine resistance [32,33]. The findings for 581gly also confirm earlier reports from the region [27]. In spite of extensive use of the drug there were surprisingly few changes in the epidemiology of *pfdhps* and *pfdhfr* mutations within these seven years.

The other mutation that seems to affect virtually all *P. falciparum* parasite samples in the region is 59arg (Table 3). This mutation is believed to modulate pyrimethamine resistance [21,34]. A study conducted in Burkino Faso, showed that *pfdhfr* 59arg, with 51ile and 108asn is an important marker for SP treatment failure [35]. Another study from Mozambique suggested that the two mutations at *pfdhfr* 59arg and *pfdhps* 437gly were enough to predict SP treatment failure [36].

The study suggests that there are two predominant *pfdhfr* mutation genotypes; 59arg + 164leu (22.58%) and 59arg + 108asn + 164leu (51.61%). There was not a single quadruple mutant (51ile + 59arg + 108asn + 164leu) in the samples, which is considered to be an indication of the highest levels of resistance to pyrimethamine [37]. Similar to the 540glu mutation in *pfdhps*, this study did not identify any sample with the 51ile mutation. However, Dokomajilar *et al.* suggested that 59arg, even in the absence of 51ile, may be more important as a marker for pyrimethamine resistance [35].

However, the results observed the presence of a sextuple mutation consisting of four *pfdhfr* (16val, 59arg, 108asn and 164leu) and two *pfdhps* (437gly, 581gly) mutations in a single individual from loggers’ camps near the border of Kalabakan to East Kalimantan. There were two sets of three *pfdhfr* and two *pfdhps* mutation (a quintuple) observed in the samples; a mutation genotype consisting of 59arg + 108asn + 164le + 437gly + 581gly, making up 41.94% of the samples and 16 val + 59arg + 164leu + 437gly + 581gly, which was found in a single sample only. The association of molecular findings with clinical treatment response was not possible due to the very low malaria prevalence and the active case detection used in this study covering a huge and largely inaccessible catchment area.

## Conclusion

Results from this study indicate that all samples harbour at least one mutation on the *pfdhfr* and *pfdhps* genes involved in SP resistance, and the predominant mutation genotype consists of a combination of 59arg + 108asn + 164le + 437gly + 581gly (41.94%). There is every indication that these genotypes confer high levels of resistance to SP in the region. This strong evidence of the high prevalence of mutations at the *pfdhfr* and *pfdhps* genes in Kalabakan highlights an urgent need for similar studies in other malaria-endemic areas in Sabah to provide urgently needed data on the current situation of SP resistance in the region.

## Competing interest

The authors declare that they have no competing interest.

## Authors’ contributions

NRA prepared study proposal and protocol, study design, lead the study in Kalabakan, data analysis and interpretation and preparation of manuscript. NAA conducted the molecular genetic studies, analysis of data, data statistics and partly drafted the manuscript. HN participated in coordinating the study, reviewing the data, data analysis and English editing. LHS, ZI, HMS oversight the project. JJ participated in the study design in the field. All authors critically reviewed the manuscript and approved the final version before submission to the Journal. All authors read and approved the final manuscript.
